# Captain Bagnold's Further Remarks

**Published:** 1817-11

**Authors:** Thomas Bagnold


*To the Editors of the London Medical and Physical Journal.*

GENTLEMEN,

SINCE writing my Observations on the Wind of a Shot,* the additional case of a lieutenant of marines, who was wounded on-board his Majesty's ship Ariadne, off Calais, in 1805, has occurred to me. I remember hearing the wound described a day or two after he received it, by the surgeon who attended him in the Royal Hospital at Deal: if I recollect rightly, a portion of the skull was removed by the ball, so as to denude the brain; yet this gentleman recovered. I am,

Your obedient Servant,

THOMAS BAGNOLD.

---

*** The above, intended by the author as an addition to his former paper, having arrived too late, is inserted in its present form.—EDIT.

* See first article of our Number for October, page 265.

